# Anaplastic lymphoma kinase L1198F and G1201E mutations identified in anaplastic thyroid cancer patients are not ligand-independent

**DOI:** 10.18632/oncotarget.14141

**Published:** 2016-12-24

**Authors:** Jikui Guan, Georg Wolfstetter, Joachim Siaw, Damini Chand, Fredrik Hugosson, Ruth H Palmer, Bengt Hallberg

**Affiliations:** ^1^ Department of Medical Biochemistry and Cell Biology, Institute of Biomedicine, Sahlgrenska Academy, University of Gothenburg, SE-405 30 Gothenburg, Sweden

**Keywords:** brigatinib, ATC, ceritinib, crizotinib, neuroblastom

## Abstract

Activating mutations in full length anaplastic lymphoma kinase (ALK) have been reported in neuroblastoma and in anaplastic thyroid cancer. ALK-L1198F and ALK-G1201E mutations were originally identified in anaplastic thyroid cancer (ATC) and characterized as constitutively activating mutations. In this study, we employed *in vitro* cell culture assays together with biochemical and *in vivo Drosophila* analyses to characterize their sensitivity to either activation by the FAM150A (AUG-β) and FAM150B (AUG-α) ALK ligands or inhibition by ALK inhibitors. Here we report that neither ALK-L1198F nor ALK-G1201E mutations result in ligand independent gain-of-function (GOF) activity in either *in vitro* biochemical analysis or the various model systems employed. ALK-L1198F is activated by the FAM150 (AUG) ligands and its ligand-dependant activity is similar to the wild type full length ALK receptor. ALK-G1201E is only very weakly activated by the FAM150 (AUG) ligands, most likely due to impaired protein stability. We conclude that neither ALK-L1198F nor ALK-G1201E displays ligand independent kinase activity, with ALK-L1198F belonging to the class of ligand dependent ALK mutations which are not constitutively active but that responds to ligand activation, while the ALK-G1201E mutation generates an unstable receptor with very low levels of kinase activity.

## INTRODUCTION

Anaplastic lymphoma kinase (ALK) belongs to the insulin receptor kinase subfamily of receptor tyrosine kinases [1], and was originally identified as a fusion protein with nucleophosmin (NPM) in anaplastic large cell lymphoma (ALCL) [2, 3]. During the last decade numerous ALK fusion proteins have been described in various cancers, such as inflammatory myofibroblastic tumors (IMT), non-small-cell lung cancer (NSCLC), diffuse large B cell lymphomas (DLBCL), renal cell carcinoma, breast cancer, colon carcinoma, serous ovarian carcinoma and oesophageal squamous cell carcinoma [1]. The full length ALK receptor possesses an extracellular ligand-binding domain, a transmembrane domain and an intracellular tyrosine kinase domain (TKD), and is activated by the FAM150A (AUG-β) and FAM150B (AUG-α) ligands [4, 5].

**Figure 1:**
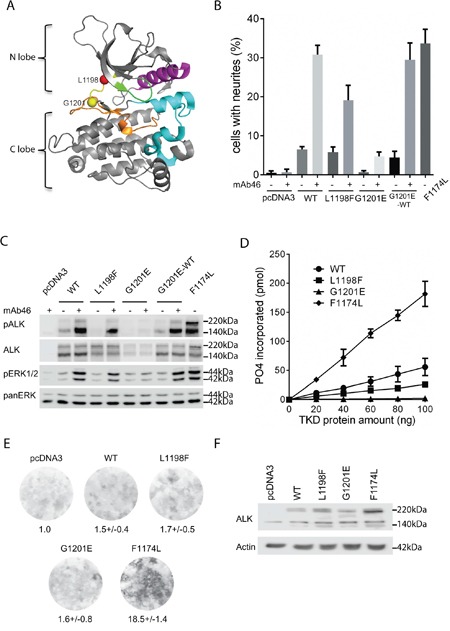
Characterization of ALK-L1198F and ALK-G1201E mutations with cell culture systems and biochemical assays **A**. Location of L1198 (red ball) and G1201 (yellow ball) residues in the hinge region (yellow) of ALK kinase domain (adapted from PDB:3LCS[19]). αC helix: magenta; glycine rich P-loop: green; activation segment: cyan; catalytic segment: orange. **B**. Neurite outgrowth of PC12 cells as a readout for ALK activity was performed with wild type ALK and ALK variants in the absence or presence of an agonist antibody against the ALK extracellular portion (mAb46). As negative control, PC12 cells alone or PC12 cells with mAb46 were employed. PC12 cells expressing activating ALK-F1174L were used as positive control. Bars represent mean percentage ± STD of neurite-carrying cells among GFP-positive cells from three independent experiments (p<0.0001, one-way ANOVA analysis). **C**. Activation of ALK with mAb46 was visualized by western blot with antibodies against phosphorylated ALK and phosphorylated ERK1/2. Total ALK and pan ERK were used as control. Blots were representative of three independent experiments. **D**. *In vitro* kinase assays with purified ALK kinase domains were performed to compare the kinase activities of wild type ALK and ALK variants. Results represent the mean value ± STD from three independent assays (p=0.0016, one-way ANOVA analysis). **E**. Representative focus formation assays for NIH 3T3 cells transfected with wild type ALK, ALK variants or empty vector. The values under the plates represent the average relative intensities ± STD from two independent experiments made in triplicates. **F**. Western blot was employed to confirm the ALK expression in NIH 3T3 cells from focus formation assay.

Point mutations in full length ALK have been observed in both familial and sporadic neuroblastoma, a common childhood cancer which arises in the tissues of the sympathetic nervous system [6–11]. Recently ALK point mutations were reported in anaplastic thyroid cancer (ATC), at residues ALK-L1198F and ALK-G1201E [12]. Most neuroblastoma mutations are situated within the kinase domain of ALK, mainly located around the α-C-helix and the activation loop. The ALK mutations found in ATC are located in the vicinity of the ATP-binding site of the kinase domain/hinge region between the N- and C-lobes (Figure 1A). ALK-positive neuroblastoma mutants fall into three classes: gain-of-function (GOF) ligand independent mutations, ligand dependent mutations which are not constitutively active and require activation with either FAM150 (AUG) ligands or agonist antibodies, and finally kinase-dead mutations [13]. Whether the ligand dependent or kinase-dead mutations are of importance in initiation and progression of disease is still unclear and will require future investigation, particularly when considering potentially complex mechanistic scenarios as the BRAF paradox [14–16]. Since the FAM150 (AUG) ligands are able to drive further activation of ALK mutants from neuroblastoma, dysregulation of the ALK ligands may potentially play a role in neuroblastoma [4].

The ALK-L1198F and ALK-G1201E mutations identified in ATC were reported to be constitutively active ALK mutations that strongly promote cell focus formation, anchorage-independent growth and cell invasion [12]. Both ALK-L1198F and ALK-G1201E activated downstream signalling, such as the PI3K/Akt and MAP kinase pathways [12]. Recently, an L1198F mutation in ALK was reported in an ALK **rearranged** NSCLC patient who first developed a crizotinib resistance mutation (ALK-C1156Y) and thereafter developed a second mutation ALK-C1156Y/L1198F, upon treatment with the third generation ALK tyrosine kinase inhibitor (TKI) lorlatinib, which results in a mutated ATP binding site that is once again crizotinib sensitive [24]. Thus further investigation of the ALK-L1198F mutation is of clinical importance not only in ATC in the context of the full length receptor but also in the EML4-ALK fusion protein involved in NSCLC.

Initially our aim was to investigate the sensitivity of ALK-L1198F and ALK-G1201E to different ALK TKIs, providing clinically relevant therapeutic information. To characterize these mutations in detail, we performed several different assays, such as neurite outgrowth and *in vitro* kinase assays as well as ectopic expression in the *Drosophila* eye. In our hands the postulated GOF mutation L1198F displays activity similar to that of wild type ALK, while the G1201E mutation results in an unstable receptor that behaves more like a kinase-dead ALK receptor.

## RESULTS

### Initial investigation of the ALK-L1198F and ALK-G1201E mutant receptors

A sensitive functional readout for receptor tyrosine kinase activity in PC12 cells is the ability of cells to induce neurite outgrowth [13]. We and others have previously shown that activation of ALK triggers differentiation of PC12 cells into sympathetic-like neurons, a process that is characterized by extension of neurites [11, 13, 17]. ALK-L1198F and ALK-G1201E have been reported as mutations with constitutive ALK tyrosine kinase activity in ATC [12]. Both residues are located in the hinge region that connects the N-terminal and C-terminal lobes of ALK kinase domain and contributes to the formation of ATP-binding site together with the glycine-rich P-loop at the interlobe cleft [18, 19] (Figure 1A). Our initial aim was to investigate whether the constitutive ALK kinase activity reported for ALK-L1198F and ALK-G1201E could be abrogated with either first or second generation ALK TKIs. However, these experiments could not be performed since neither ALK-L1198F nor ALK-G1201E were able to generate neurite outgrowth when expressed in PC12 cells (Figure 1B). This is in contrast to the ALK-F1174L positive control, which is a well characterised GOF ALK neuroblastoma mutation [7, 8], that induced robust neurite outgrowth (Figure 1B). Stimulation of ALK-L1198F and ALK-G1201E with an agonist antibody (mAb46) [20] led to neurite outgrowth, however, less neurite outgrowth was observed when compared to wild type ALK and the ALK-F1174L positive control (Figure 1B). Consistent with the neurite outgrowth results, stimulation of ALK-WT and ALK-L1198F led to ALK phosphorylation and activation of downstream MAPK/ERK signaling (Figure 1C). While ALK-L1198F displayed reduced levels of both ALK and ERK1/2 activation upon stimulation when compared to ALK-WT, stimulation of ALK-G1201E did not result in any detectable activity (Figure 1C). Moreover, both ALK-L1198F and ALK-G1201E failed to generate focus formation in a NIH 3T3 transformation assay (Figure 1E). In contrast, ALK-F1174L displayed robust focus formation (Figure 1E). The presence of ALK proteins in NIH3T3 cells during the focus formation assay was confirmed by western blot (Figure 1F). These results indicate that neither ALK-L1198F nor ALK-G1201E is ligand-independent GOF mutant in our hands. *In vitro* kinase assays were performed as a complement to these analyses. ALK-F1174L displayed the highest tyrosine kinase activity - almost 4 times that of wild type ALK (Figure 1D). In contrast, ALK-L1198F kinase activity was 50% of wild type ALK, while ALK-G1201E did not show any notable kinase activity (Figure 1D). The kinase assay results obtained are consistent with observations in PC12 and NIH 3T3 cells (Figure 1B and 1C). Taken together, our data strongly suggests that both ALK-L1198F and ALK-G1201E lead to impaired ALK activity and are not ligand-independent GOF mutations.

### FAM150A (AUG-β) and FAM150B (AUG-α) activate the ALK-L1198F and ALK-G1201E mutants

**Figure 2:**
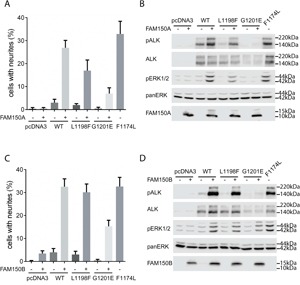
Activation of ALK-L1198F and ALK-G1201E by ALK ligands FAM150A (AUG-β) and FAM150B (AUG-α) **A**. Neurite outgrowth was measured in PC12 cells co-expressing ALK-WT, ALK-L1198F, or ALK-G1201E with FAM150A (AUG-β). As negative control, PC12 cells alone or PC12 cells expressing FAM150A (AUG-β) alone were employed. PC12 cells expressing activating ALK-F1174L were used as positive control. Bars represent the mean percentage ± STD of neurite-carrying cells among GFP-positive cells from three independent experiments (p<0.0001, one-way ANOVA analysis). **B**. Activation of ALK with FAM150A (AUG-β) was visualized by western blot with antibodies against phosphorylated ALK and phosphorylated ERK1/2. Total ALK and pan ERK were employed as control. The presence of FAM150A (AUG-β) was also detected. Blots were representative of three independent experiments. **C**. Neurite outgrowth was measured in PC12 cells co-expressing ALK-WT, ALK-L1198F, or ALK-G1201E with FAM150B (AUG-α). As negative control, PC12 cells alone or PC12 cells expressing FAM150B (AUG-α) alone were employed. PC12 cells expressing activating ALK-F1174L were used as positive control. Bars represent the mean percentage ± STD of neurite-carrying cells among GFP-positive cells from three independent experiments (p<0.0001, one-way ANOVA analysis). **D**. Activation of ALK with FAM150B (AUG-α) was visualized by western blot with antibodies against phosphorylated ALK and phosphorylated ERK1/2. Total ALK and pan ERK were employed as control. The presence of FAM150B (AUG-α) was also confirmed. Blots were representative of three independent experiments.

We next examined whether ALK-L1198F and ALK-G1201E were activated/phosphorylated upon stimulation with the FAM150A (AUG-β) and FAM150B (AUG-α) ligands. In the absence of stimulation neither ALK-L1198F nor ALK-G1201E gave rise to neurite outgrowth upon expression in PC12 cells (Figures 2A and 2C). However, upon stimulation with FAM150A (AUG-β) or FAM150B (AUG-α) ligands both ALK-L1198F and ALK-G1201E were able to mediate neurite outgrowth, although at levels less than those observed with wild type ALK or the constitutively active ALK-F1174L neuroblastoma mutant (Figure 2A and 2C). While both the ALK-F1174L positive control and wild type ALK were phosphorylated upon stimulation with either FAM150A (AUG-β) or FAM150B (AUG-α) (Figure 2B and 2D), stimulation of ALK-L1198F led to reduced levels of ALK Y1604 phosphorylation when compared with wild type ALK, while ALK-G1201E Y1604 phosphorylation was barely detectable (Figure 2B and 2D). In agreement with the levels of ALK Y1604 phosphorylation of the ALK-G1201E mutant, we were unable to detect activation of downstream ALK targets such as ERK1/2 with FAM150A (Figure 2B), although low levels of ERK activation were seen in response to FAM150B (Figure 2D). We also observed that the ALK-G1201E mutant was expressed at reduced levels when compared with either wild type ALK or the ALK-F1174L and ALK-L1198F mutants. In contrast, the ALK-F1174L positive control activates MAPK/ERK signalling even in the absence of ligand (Figure 2B and 2D). Upon stimulation with the either FAM150A (AUG-β) or FAM150B (AUG-α) wild type ALK mediated ERK1/2 phosphorylation. The remaining ALK ATC mutation - ALK-L1198F - did result in activation of ERK1/2 upon stimulation with FAM150A (AUG-β) or FAM150B (AUG-α), although stimulation was not as robust as that observed with either wild type ALK or the ALK-F1174L mutant (Figure 2B and 2D).

Given our consistent observation of low levels of expression of the ALK-G1201E mutant we decided to investigate this further. Firstly, both ALK-L1198F and ALK-G1201E expression constructs were confirmed by sequencing to exclude any possibilities that an error had occurred during construction. We next mutated the ALK-G1201E mutant back to wild type. This new revertant wild type ALK was once again stably expressed when compared with the ALK-G1201E mutant (Figure 1C). Moreover, upon stimulation with mAb46 the wild type that had been reverted from ALK-G1201E re-acquired the ability to become phosphorylated and to activate downstream ERK1/2 again (Figure 1C) and therefore to give neurite outgrowth (Figure 1B). These data suggest that the ALK-G1201E mutation generates an unstable protein that displays very low levels of kinase activity. Based on these results we hypothesized that substitution of G1201 to an amino acid with a bulkier side chain may destabilize the protein thereby resulting in reduced kinase activity, reflecting a structural constraint at residue 1201 for amino acids with smaller side chain. To test this hypothesis, we generated ALK-G1201R and ALK-G1201A mutations (Supplementary Figure 1). In keeping with our hypothesis, the ALK-G1201R mutation was poorly expressed and exhibited impaired kinase activity when compared with wild type ALK. In contrast, the ALK-G1201A mutation was well expressed and responded to stimulation with the agonist antibody mAb46 in a manner similar to wild type ALK (Supplementary Figure 1).

Taken together, our results indicate that neither of the ATC ALK-L1198F/ALK-G1201E mutations is as active as wild type ALK when stimulated with either FAM150A (AUG-β) or FAM150B (AUG-α). Further, the ALK-G1201E protein appears to be intrinsically unstable. Thus, we are unable to produce evidence that either ALK-L1198F or ALK-G1201E is a GOF mutation in ALK.

### Examination of ALK-L1198F and ALK-G1201E activity in the *Drosophila* eye

**Figure 3:**
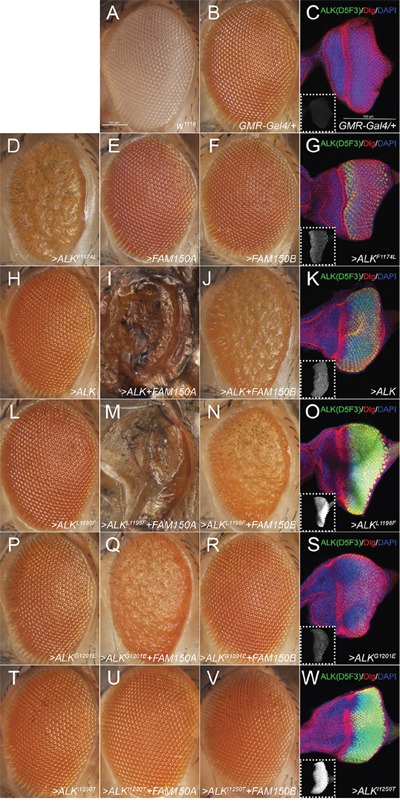
Effects of the ectopic expression of human ALK variants in the *Drosophila* eye **A**. ‘wild type’ *white^1118^* (*w^1118^*) and **B, C**. heterozygous *GMR-Gal4* (*GMR-Gal4>/+*) driver controls. **D-W**. Heterozygous *GMR-Gal4* drives expression of one copy of each *UAS*-transgene (>) indicated. Eye morphology is not affected upon expression of FAM150A (AUG-β; E), FAM150B (AUG-α; F) or wild type ALK (H) alone, ALK-L1198F (L), ALK-G1201E (P), or the kinase-dead variant ALK-I1250T (T). However, ectopic expression of the constitutively active ALK-F1174L variant severely interferes with normal eye development (D). Simultaneous expression of the ALK activating ligands FAM150A (AUG-β) and FAM150B (AUG-α) together with ALK-WT or ALK-L1198F disrupts eye morphology in a similar way (J, N) and even induces lethality (I, M) whereas co-expression with ALK-G1201E or ALK-I1250T induces a milder (Q) or no phenotype (R, U, V). (C, G, K, O, S, W) Staining of third instar larval eye discs with the ALK antibody D5F3 (green, white in smaller insets), anti-discs large (Dlg, red) and DAPI (blue) reveals that ectopic expression of ALK-G1201E (S) results in a considerably lower amount of detectable ALK protein than ALK-L1198F (O). Comparably low expression levels are observed in case of the functional ALK-WT (K) and ALK-F1174L (G) transgenes whereas high levels of ALK-I1250T (W) do not effect eye development. Confocal stacks (14 μm) of G, K, O, S, W were acquired with the same laser intensity, PMT- and pinhole settings. Scale bars are 100 μm.

Finally we employed ectopic expression of ALK-L1198F and ALK-G1201E in the *Drosophila* model to challenge the ATC mutations. *Drosophila melanogaster* has been successfully employed as an *in vivo* model to characterize human ALK mutations, since this system provides a very clean background [11, 13]. The *Drosophila* Alk ligand, Jelly Belly (Jeb), is unable to activate either human or mouse ALK orthologues [11, 13, 21, 22], however, co-expression with the human ALK ligands FAM150A (AUG-β) or FAM150B (AUG-α) provides robust activation of the human receptor in the *Drosophila* eye [4] (Figure 3I and 3J). To further characterize the activating potential of the human ALK-L1198F and ALK-G1201E mutations, we generated transgenic fly lines and employed the *GMR-Gal4* driver to direct ectopic protein expression in developing photoreceptors (Figure 3L and 3P). As further controls we employed transgenic flies expressing either constitutively active ALK-F1174L (Figure 3D), ligand-dependent wild type ALK (Figure 3H) or ‘kinase dead’ ALK-I1250T [23] (Figure 3T). As observed previously, expression of the well-characterized, ligand-independent ALK-F1174L allele, which encodes for a constitutively active receptor [11], resulted in a severe rough eye phenotype (Figure 3D). A similar phenotype is also found in flies ectopically expressing other *ALK* GOF alleles [13, 17], whereas expression of human FAM150A (AUG-β), FAM150B (AUG-α), ALK-WT, ALK-L1198F or ALK-G1201E alone (Figure 3E, 3F, 3H, 3L and 3P) did not reveal any obvious phenotype in adult flies when compared to the *w^1118^* and GMR driver controls (Figures 3A and 3B). The lack of activity of either human ALK-L1198F or ALK-G1201E mutation in the fly eyes strongly supports the conclusion that they are not GOF in nature but in fact represent ligand-dependent or even loss-of-function (LOF) alleles.

To address this we co-expressed the previously reported activating ALK ligands FAM150A (AUG-β) or FAM150B (AUG-α) together with either ALK-L1198F or ALK-G1201E in the developing eye. In case of ALK-L1198F we observed a severe rough eye phenotype upon activation with FAM150B (AUG-α) (Figure 3N). Activation with FAM150A (AUG-β) not only led to a rough eye phenotype but also induced pupal lethality (Figure 3M). These effects were indistinguishable from those observed in flies expressing FAM150 (AUG) ligands together with ALK-WT (Figures 3I and 3J) suggesting that ALK-L1198F encodes a ligand-dependent, inducible form of the receptor. Surprisingly, co-expression of FAM150B (AUG-α) and ALK-G1201E (Figure 3R) resulted in no obvious defects, resembling the previously characterised non-inducible, ‘kinase-dead’ mutation ALK-I1250T (Figures 3U and 3V). Moreover, combined expression of FAM150A (AUG-β) and ALK-G1201E (Figure 3Q) no longer resulted in lethality but induced a rough eye phenotype suggesting that the ALK-G1201E mutation, while not completely kinase dead, is in fact a LOF allele. Complementary immunofluorescence analysis clearly showed that while ALK-L1198F and ALK-I1250T were strongly expressed in the eye discs of *Drosophila* third instar larvae (Figures 3O and 3W), the amount of detectable ALK protein was much lower in ALK-G1201E expressing eye discs (Figure 3S), in keeping with the results in our mammalian cell expression above. Since the functional ALK-F1174L and ALK-WT transgenes also exhibited lower expression levels than ALK-L1198F (compare Figure 3G, 3K to 3O) it is conceivable that ALK-G1201E encodes for a receptor with impaired function. Taken together, our *Drosophila* analyses also suggest that neither ALK-L1198F nor ALK-G1201E is a ligand independent GOF ALK mutant.

### Sensitivity of ALK-L1198F to ALK inhibitors

**Figure 4:**
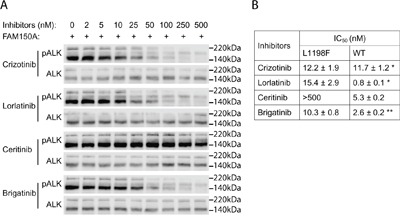
Inhibition profiling of ALK TKIs on ALK-L1198F **A**. PC12 cells expressing ALK-L1198F were treated with serial dilution of ALK inhibitors as indicated and stimulated with FAM150A (AUG-β) ligand. Phosphorylation of ALK (Y1604) was detected with pALK antibody and total ALK was used as loading control. Blots were representative of three independent experiments. Half maximal inhibitory concentrations (IC50s) of different ALK inhibitors are shown in **B**. *Data cited from [25]. **Data from [39].

**Figure 5:**
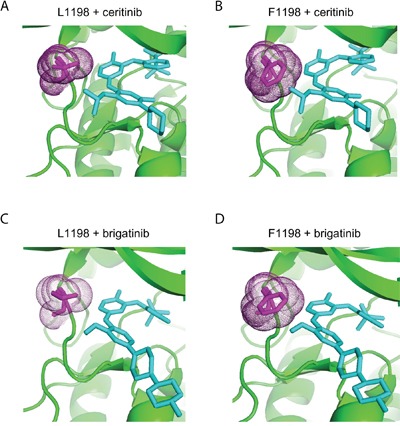
Structural modelling of ceritinib and brigatinib binding to ALK-WT and ALK-L1198F ALK wild type (A and C) structure and mutant ALK-L1198F structure (B and D) generated in PyMol binding to ceritinib (PDB;4MKC) (A and B), and brigatinib (PDB;5J7H) (C and D). Residue 1198 is shown in magenta with dots. Ceritinb and brigatinib are indicated in figure and shown in cyan.

The ALK-L1198F mutation in the context of the EML4-ALK fusion oncogene was recently reported in a NSCLC patient treated with ALK inhibitors. The ALK-L1198F that arose in this patient in response to treatment with the third generation ALK TKI, lorlatinib, was shown to be sensitive to inhibition by crizotinib [24]. Since ALK-L1198F does not behave as a GOF mutation in the full length ALK receptor in our hands, we therefore examined the ability of different inhibitors to abrogate its activity in PC12 cells in the presence or absence of ligand stimulation. As readout for ALK activity we employed phosphorylation of ALK Y1604, which reflects ALK activation [13]. We clearly observed that full length ALK-L1198F mutation was more resistant than wild type ALK to lorlatinib inhibition as measured by Y1604 phosphorylation. The IC50 of lorlatinib for ALK-L1198F was 15.4 ± 2.9 nM, approximately 20 times of that for wild type ALK [25]; however the L1198F mutation did not affect the ability of crizotinib to abrogate ALK activity in this assay (Figures 4A and 4B). The ALK-L1198F mutation caused a mild resistance to another next-generation ALK TKI brigatinib (Figures 4A and 4B). The FDA-approved second-generation ALK TKI ceritinib was completely unable to inhibit the activity of ALK-L1198F in response to FAM150 (AUG) ligand activation, since no inhibition of ALK Y1604 phosphorylation was observed even with 500 nM of ceritinib. The observed IC50 values of ALK-L1198F for brigantinib, crizotinib and lorlatinib were in a similar range (10.3 nM to 15.4 nM) (Figures 4A and 4B). Taken together, our results and those recently reported for the EML4-ALK fusion in NSCLC [24] indicate that ceritinib is not an option for treatment of patients harbouring the L1198F mutation in either the full length ALK or the EML4-ALK fusion protein. The L1198F mutation in full length ALK probably mediates a steric clash between the phenylalanine residue and ceritinib in a similar manner seen when modelling lorlatinib in the EML4-ALK C1156Y-L1198F mutant [24] (Figure 5B). As a read out, the ALK-L1198F-ceritinib interaction increases the half maximal inhibitory ceritinib concentration from 5.3 nM to more than 500 nM (Figure 4B). Modelling of brigatinib (Figures 5C and 5D), lorlatinib [24] and crizotinib [24] to ALK does not reveal any obvious steric hindrance. However, investigation of the EML4-ALK-L1198F mutation has been reported to show a more favourable binding to crizotinib relative to wild type EML4-ALK [24]

## DISCUSSION

Taken together, the results presented here clearly show that ALK-L1198F and ALK-G1201E are not GOF mutations in the context of full length ALK. Furthermore, mutation of G1201E results in an unstable protein with dramatically reduced kinase activity. The observed problems with protein stability of the ALK-G1201E mutant presents a challenge in addressing whether its low level of activity is a result of reduced kinase activity or protein levels or a combination of both. Clearly reversion of ALK-G1201E to ALK-E1201G results in a protein that is expressed and can be stimulated with ligands, such as agonist antibody or ligands FAM150A and B. Further, the hypothesis that substitution of G1201 to an amino acid with a bulkier side chain, such as glutamate or arginine may destabilize the protein drastically impacting on kinase activity is supported by our data. Thus, our analyses suggest structural constraints at residue 1201 in ALK. Replacing the G1201 with another bulky residue, arginine in this case, also results in an unstable protein. In contrast, replacing G1201 with alanine results in a change of stability or kinase activity to levels comparable with wild type ALK.

Based on our findings, the involvement of ALK activating mutations in the context of the full length ALK receptor in thyroid cancer should be carefully re-evaluated. In contrast to the single report of activating ALK mutations (ALK-L1198F and ALK-G1201E) in the full length receptor [12], a number of articles have reported ALK fusion genes in thyroid cancer, where they appear to represent a small fraction of papillary thyroid carcinoma (PTC). Fusion partners involved include EML4, STRN, TFG, and GTF2IRD1 [26–32]. ALK fusions have also been descried in medullary thyroid cancer (EML4-ALK and Glutamine:fructose-6-phosphate transaminase 1 (GFPT1)-ALK), with patient response to crizotinib reported in an EML4-ALK patient [33].

Both clinical and preclinical data support ALK fusion proteins are drivers in tumorigenesis [34], where oligomerisation mediated by the ALK fusion partner serves to dimerize and activate the ALK kinase domain. The appearance of clinical TKI-resistant ALK mutations, which are mostly clustered around the ATP/TKI binding site in the kinase domain result in unfavourable binding for the specific ALK TKIs employed. The ALK-L1198F mutation in the context of the EML4-ALK fusion protein leads to a dramatically reduced ability of this mutant to be inhibited by ceritinib as compared to wild type EML4-ALK [24]. However, the oncogenic activity of the EML4-ALK-L1198F mutant is still due to oligomerisation and activation driven by the EML4 fusion partner. In contrast, mutation of L1198F in the context of the full length receptor does not result in ligand independent ALK activation.

The COSMIC database reports ALK sequencing results for 720 thyroid cancer samples, identifying 12 samples with mutations, including ALK-L1198F and ALK-G1201E. While it is possible that some of the other candidate mutations may be activating in nature they have not been tested and none overlap with mutations shown to activate ALK in neuroblastoma [35]. Whether cellular context, in a thyroid cell environment, plays a role in the *in vivo* activity of these ALK mutations remains to be determined. In conclusion, while mutations in full length ALK leading to activation of the receptor are well characterised in neuroblastoma and supported by a substantial body of work, the activation of full length ALK in thyroid cancer requires further critical evaluation. Finally, ceritinib may not be an option for treatment of patients harboring the L1198F mutations in either the full-length ALK or the EML4-ALK fusion protein.

## MATERIALS AND METHODS

### Generation of human ALK mutant constructs

ALK-L1198F, ALK-G1201E and other ALK-G1201 mutation constructs were generated based from pcDNA3-ALK-WT (NM_004304.3) by Eurofins Genomics. ALK-F1174L has been described previously [11]. ALK-G1201E→WT was generated with QuickChange Site-Directed Mutagenesis Kit (Stratagene) in which the G1201E mutation was mutated back to wild type. All the mutations generated in the kinase domain were confirmed by sequencing from both directions.

### Neurite outgrowth assay

PC12 cells (2×10^6^) were transfected by electroporation in an Amaxa electroporator, using 0.75 μg of ALK constructs and 0.5 μg of pEGFP-C1 (Clontech) and 100 μL of Ingenio electroporation solution (Mirrus Bio LCC). After transfection, cells were transferred to DMEM supplemented with 7% horse serum and 3% FBS and seeded into 24-well plates. An agonist monoclonal antibody mAb46 was added at 1 μg/mL to activate the ectopically expressed ALK receptors. Two days after transfection, the percentage of GFP-positive and neurite-carrying cells versus GFP-positive cells was estimated under a Zeiss Axiovert 40 CFL microscope. To be judged as a neurite-carrying cell, the neurites of the cell had to reach at least twice the length of the cell body. Experiments were performed in triplicates and each sample within an experiment was assayed in duplicate.

### Cell culture, lysis, and immunoblotting

PC12 cells were transfected with different ALK constructs by electroporation. Cells were serum-starved for 36 hours prior to stimulation with 1 μg/ml of the activating mAb46 for 30 minutes Cells were washed with ice-cold PBS prior to harvest in lysis buffer [25 mM of Tris-Cl, pH7.5, 150 mM of NaCl, 1% (v/v) Triton X-100, 1 mM of DTT, protease inhibitor cocktail tablet (Roche)]. Cell lysates were cleared by centrifugation at 14,000 rpm for 15 minutes at 4°C. Samples were boiled in 1x SDS sample buffer and analyzed by immunoblotting. Primary antibodies used for immunoblotting were: anti-pan-ERK (1:10,000) from BD Transduction Laboratories (Franklin Lakes, NJ); anti-pALK (Y1604) and anti-pERK1/2 (T202/Y204) from Cell Signaling Technology (Danvers, MA); anti-FAM150A and anti-FAM150B antibodies from Atlas Antibodies (Stockholm). Monoclonal antibody 135 (anti-ALK) was produced in the Hallberg laboratory against the extracellular domain of ALK as described [20]. Horseradish-peroxidase-conjugated secondary antibodies goat anti-rabbit IgG and goat anti-mouse IgG (1:5,000) were from Thermo Scientific (Waltham, MA).

### Stimulation with ALK ligands FAM150A (AUG-β) and FAM150B (AUG-α)

For neurite outgrowth assays, PC12 cells (2×10^6^) were transfected by electroporation in an Amaxa electroporator, using 0.3 μg of pcDNA3 vector or different ALK constructs, together with 0.5 μg of pEGFP-C1, and with or without 1 μg of either FAM150A (AUG-β) or FAM150B (AUG-α) plasmid and 100 μL of Ingenio electroporation solution (Mirrus Bio LCC). After transfection, cells were transferred to DMEM supplemented with 7% horse serum and 3% FBS and seeded into 24-well plates. After twenty four hours, cells were analysed as described above. PC12 cells transfected with ALK-F1174L alone were used as control.

For immunoblotting, PC12 cells (2×10^6^) were electroporated with 0.3 μg of pcDNA3 or different ALK constructs, together with or without 1 μg of either FAM150A (AUG-β) and FAM150B (AUG-α) plasmid. PC12 cells electroporated with ALK-F1174L alone were used as positive control. Twenty-four hours after transfection, cells were starved for another 30-36 hours prior to lysis. Cells were then lysed and analysed with immunoblotting as described above.

### Generation of recombinant ALK TKD proteins

DNA encoding ALK residues 1090-1416 which includes the whole tyrosine kinase domain (TKD) was amplified from different ALK constructs with Phusion High-Fidelity DNA polymerase (ThermoScientific) and cloned into pFastBac/NT-TOPO vector (Invitrogen) for expression of 6× histidine-tagged recombinant ALK TKD proteins in Sf21 cells. Recombinant baculovirus was generated using the Bac-to-Bac baculovirus expression system (Invitrogen). Sf21 cells were infected with P2 virus stock for 3 days at 27°C and then lysed in native Ni-NTA lysis buffer (50 mM NaH_2_PO_4_ pH8.0, 300 mM NaCl, 10 mM imidazole, 1% Triton X-100 and protease inhibitor cocktail). After centrifugation, His-tagged TKD proteins were recovered from the lysis supernatant using Ni-NTA His·Bind resins (Novagen). After two washes, the TKD proteins bound to resins were incubated in 50 mM Tris-Cl pH 7.4, 150 mM NaCl, 10 mM MgCl_2_ and 2 mM ATP at 30°C for 1 hour for further ALK TKD autophosphorylation. After two additional washes, ALK TKD proteins were eluted and their concentrations were determined by absorbance at 280 nm. Purity of ALK TKD proteins was assessed by SDS-PAGE and Coomassie blue staining.

### *In vitro* kinase assays

Analysis of substrate phosphorylation by ALK TKD employed a peptide mimic of the ALK activation loop with sequence: ARDIYRASYYRKGGCAMLPVK (Caslo, Danmark), referred to as YYY peptide [36]. Assays were performed using radiolabeled ATP as described previously [37]. Different amount of ALK TKD proteins were used with fixed concentration of ATP and peptide at 0.1 mM and 0.2 mM respectively. Assays were conducted at 30°C for 30 minutes and the incorporation of ^32^P from γ-^32^P ATP into the substrate was detected with Wallac 1410 Liquid Scintillation Counter (GMI). Assays were performed three times independently and each sample within an assay was set in duplicate.

### Transformation assay

NIH 3T3 cells (4.5×10^4^) were seeded in collagen-coated 12-well plates prior to transfection for 6 hours with 1.75 μg of pcDNA3 vector, pcDNA3-ALK-WT, pcDNA3-ALK-L1198F, pcDNA3-ALK-G1201E or pcDNA3-ALK-F1174L together with 5 μl of Lipofectamine 2000 (Invitrogen) in 0.3 ml Opti-MEM. The next day, 60% of the cells were transferred to six-well plates and maintained in DMEM with 10% heat-inactivated FBS and 0.5 mg/ml G418 until the cells reached confluency. Then, the cells were cultivated in DMEM with 5% heat-inactivated FBS and 0.25 mg/ml G418 for approximately 10 days. The cells were then fixed using methanol and stained with 0.25% crystal violet. The focus intensities were subsequently analysed [17].

### Expression of human ALK constructs in the *Drosophila* eye

Standard *Drosophila* husbandry procedures were followed. *Stocks* were maintained on a potato-mash based diet at room temperature. Crosses were performed at 25°C and 60% humidity level. The *UAS-ALK-L1198F* and *UAS-ALK-G1201E* constructs were generated using standard cloning techniques and transgenic flies were obtained by injection (BestGene Inc.). *white^1118^* (*w^1118^*) flies were used as control; *GMR-Gal4* (#1104) was received from the Bloomington *Drosophila* Stock Center at Indiana University (BDSC; NIH P40OD018537). *UAS-ALK*, *UAS-ALK-F1174L*, *UAS-ALK-I1250T* as well as *UAS-FAM150A (AUG-β)* and *UAS-FAM150B (AUG-α)* were used for ectopic expression studies in the developing *Drosophila* eye employing the *GAL4-UAS* system. Staining of imaginal discs was described previously [38]. The rabbit monoclonal ALK (D5F3) antibody was purchased from Cell Signalling and employed at a 1:200 dilution. The hybridoma, monoclonal mouse antibody 4F3 (anti-discs large; employed at 1:500), deposited by Corey Goodman was obtained from the Developmental Studies Hybridoma Bank (DSHB), created by the NICHD of the NIH and maintained at The University of Iowa. Samples were analysed under Zeiss Axio Imager.Z2 and AxioZoom.V16 microscopes. Images were acquired with a Zeiss LSM800 confocal microscope or with an Axiocam 503 colour camera employing ZEN blue edition software.

### ALK inhibitor profiles on ALK-L1198F mutation

PC12 cells (2×10^6^) were transfected by electroporation in an Amaxa electroporator using 1.5 μg of ALK L1198F construct and 100 μL of Ingenio electroporation solution (Mirrus Bio LCC). Cells from three electroporations were pooled together, mixed and equally seeded into 9 wells of one 24-well plate. After 24-36 hours culture, cells were treated with serial dilutions of the indicated inhibitors for 1.5 hours before being stimulated with FAM150A (AUG-β) for another 30 minutes. Cells were washed with cold 1X PBS and lysed with 1X SDS sample buffer and samples were boiled at 95°C for 5 minutes. Phospho-ALK (Y1604) antibody was used to detect ALK phosphorylation and ALK mAb135 was used to detect total ALK. The intensity of pALK (Y1604) and total ALK bands was quantified with Image Studio Lite 3.1 software. Data were normalised to the 0 nM inhibitor samples. GraphPad Prism 6.0 was used to calculate IC50 values by fitting data to a log (inhibitor concentration) vs. normalised response (variable slope) equation.

## SUPPLEMENTARY MATERIALS FIGURES


