# Supplementary material for: Anaplastic lymphoma kinase L1198F and G1201E mutations identified in anaplastic thyroid cancer patients are not ligand-independent
Source: Oncotarget. 2016 Dec 24;8(7):11566–78. doi: 10.18632/oncotarget.14141 (PMC5355286; doi:10.18632/oncotarget.14141)
Supplement: Supplementary file 1 [file oncotarget-08-11566-s001.pdf]

Anaplastic lymphoma kinase L1198F and G1201E mutations identified in anaplastic thyroid cancer patients are not ligand-independent

SUPPLEMENTARY METHODS

PC12 cells (2×10^6) were electroporated with 0.3 µg of different ALK constructs in an Amaxa electroporator. PC12 cells electroporated with pcDNA3 vector or ALK-F1174L alone were used as negative or positive control respectively. Twenty-four hours later, cells were serum-starved for 36 hours prior to stimulation with 1 µg/ml of the activating mAb46 for 30 minutes as indicated in the figure. Cells were then lysed and analysed with immunoblotting as described in the main text.

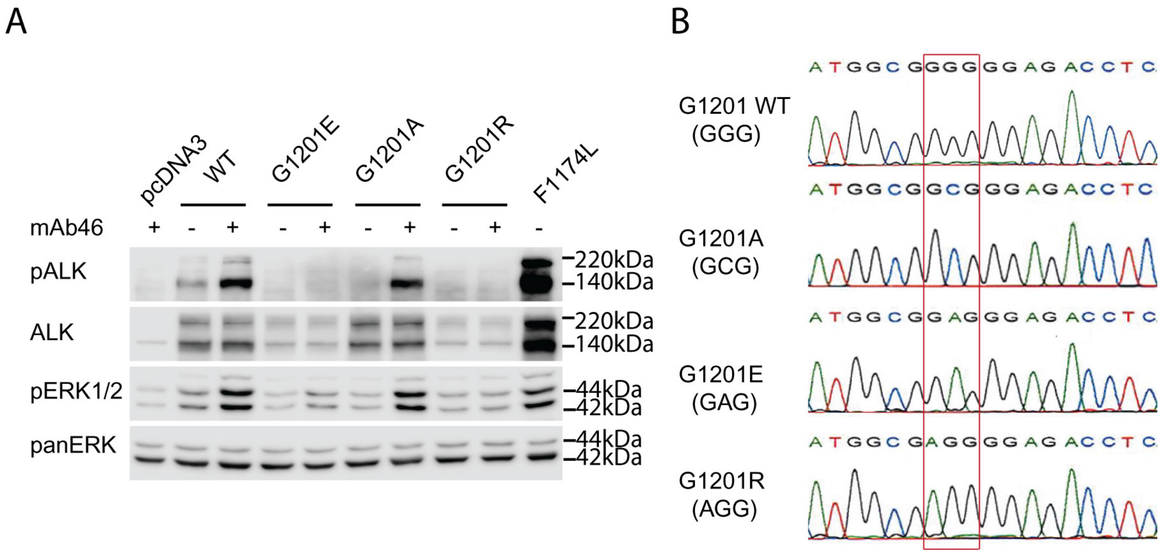

Supplementary Figure 1: A. Activation of different G1201 variants and downstream ERK1/2 by agonist antibody mAb46. PC12 cells transferred with pcDNA3 vector and ALK F1174L were used as negative and positive controls respectively, and wid type ALK was used to compare with different G1201 variants. **B.** Confirmation of the G1201 variants (red box) by Sanger sequencing.
